# A sub‐national HIV epidemic appraisal in Kenya: a new approach for identifying priority geographies, populations and programmes for optimizing coverage for HIV prevention

**DOI:** 10.1002/jia2.26245

**Published:** 2024-07-10

**Authors:** Ramesh Banadakoppa Manjappa, Parinita Bhattacharjee, Souradet Yuh‐Nan Shaw, Joshua Gitonga, Japheth Kioko, Franklin Songok, Faran Emmanuel, Peter Arimi, Helgar Musyoki, Ruth Laibon Masha, James Blanchard

**Affiliations:** ^1^ Institute for Global Public Health University of Manitoba Winnipeg Manitoba Canada; ^2^ Partners for Health and Development in Africa Nairobi Kenya; ^3^ National Syndemic Diseases Control Council Ministry of Health Nairobi Kenya; ^4^ National AIDS and STI Control Programme Ministry of Health Nairobi Kenya; ^5^ Global Fund to Fight AIDS, Tuberculosis and Malaria Geneva Switzerland

**Keywords:** HIV epidemiology, HIV prevention, HIV epidemic appraisal, sub‐national

## Abstract

**Introduction:**

The HIV Prevention 2025 Roadmap, developed by UNAIDS, recommends the adoption of a precision prevention approach focused on priority populations and geographies. With reduction in new HIV acquisitions in many countries, designing a differentiated HIV prevention response, using a Programme Science approach, based on the understanding of the epidemic and transmission dynamics at a sub‐national level, is critical.

**Methods:**

To support strategic planning, an epidemic appraisal at the sub‐national level across 47 counties, with the 2019 population ranging from 0.14 million in Lamu to 4.40 million in Nairobi City, was conducted in Kenya using several existing data sources. Using 2021 Spectrum/EPP/Naomi model estimates of national and sub‐national HIV incidence and prevalence, counties with high HIV incidence and prevalence were identified for geographic prioritization. The size of local key population (KP) networks and HIV prevalence in key and general populations were used to define epidemic typology and prioritize populations for HIV prevention programmes. Analysis of routine programme monitoring data for 2021 was used to assess coverage gaps in HIV prevention programmes, including prevention of vertical transmission, anti‐retroviral therapy, KP programmes, adolescent girls and young women programme, and voluntary male medical circumcision programme.

**Results:**

Ten counties with more than 1000 incident acquisitions in 2021 accounted for 57% of new acquisitions. Twenty‐four counties were grouped into the concentrated epidemic type—due to their low prevalence in the general population, high prevalence in KPs and relatively higher density of female sex workers and men who have sex with men populations. Four counties reflected a generalized epidemic, where HIV prevalence was more than 10% and 30%, respectively, among the general and key populations. The remaining 19 counties were classified as having mixed epidemics. Gaps in programmes were identified and counties where these gaps need to be addressed were also prioritized.

**Conclusions:**

The HIV burden in Kenya is unevenly distributed and hence the mix of prevention strategies may vary according to the epidemic typology of the county. Prioritization of programmes based not only on disease burden and epidemic typology, but also on the prevailing gaps in coverage for reducing inequities is a key aspect of this appraisal.

## 1 INTRODUCTION

The HIV prevention field has seen a rapid evolution over the last decade. Numerous programmes to prevent HIV acquisition are now available; however, these programmes have not been implemented and utilized in relation to the magnitude of the HIV burden globally. For example, the Joint United Nations Programme on HIV/AIDS (UNAIDS) and the World Health Organization both recommend that countries invest strategically in scaling up new preventive programmes such as oral pre‐exposure prophylaxis as a part of combination of HIV prevention approaches [1, 2]. They also recommend to simultaneously revitalize long‐established and proven approaches, such as the promotion of condoms, voluntary medical male circumcision (VMMC), retention of people living with HIV on anti‐retroviral therapy (ART), addressing social and legal barriers that impede many people from accessing HIV services, and eliminating stigma and discrimination, especially in healthcare settings. The UNAIDS HIV Prevention Roadmap 2025's ten‐point action plan recommends countries adopt a “precision prevention approach” focused on key and priority populations and geographies to develop national HIV prevention goals aligned to 2025 targets [3]. Increasingly, countries need to deliver these prevention services more efficiently to reach more people using an equity lens, at a lower cost, including integrating HIV prevention into sexual and reproductive health services and other healthcare platforms [4].

Given heterogeneity in HIV epidemic trajectories globally, understanding changing local epidemiological dynamics is important for national HIV prevention policies, and to inform the implementation of efficient responses [5]. Improved understanding of the population size and organizational typologies of sex work is also considered central to a country's planning process for scaling up focused HIV prevention programmes [6]. A focused approach that prioritizes people and locations at greater risk of HIV acquisition and adapts programming to reflect local epidemiological context is more likely to increase the efficiency and effectiveness of HIV prevention investments [7, 8]. The static Modes of Transmission metric is considered to have limited value in guiding the prioritization of HIV prevention targets, particularly since it underestimates the contribution of epidemic drivers to HIV transmission over time [9]. Approaches that yield accurate and timely guidance on the status and drivers of ongoing local transmission, individual dynamism and epidemic trajectory are critical to address the key behaviours that drive HIV transmission. HIV epidemic appraisals that characterize heterogeneity and inequities in the context of the HIV epidemic and the response fulfil this role, enabling national and sub‐national governments to invest in longer‐term strategies to reduce the incidence and prevalence of HIV [5, 9].

### 1.1 The Kenya context

In 2019, Kenya had a population of 47.6 million, and the population across 47 counties ranged from 0.14 million in Lamu to 4.40 million in Nairobi City [10]. In 2022, Kenya ranked 11 in the world in terms of HIV epidemic, reporting a prevalence of 3.7% [11]. The annual HIV incidence was 15 per 1000 among adult women and 13 per 1000 among adult men (15−64 years) in 2018 [12, 13]. Kenya has a devolved governance system comprising the national government and 47 county (i.e. sub‐national) governments that are autonomous and responsible for managing health facilities and pharmacies, and promotion and provision of healthcare services for HIV, tuberculosis, malaria and reproductive maternal, neonatal, child and adolescent health [14].

In 2014, the government of Kenya committed to prioritizing and scaling up HIV prevention programmes with the development of the Kenya HIV Prevention Revolution Roadmap 2030 [15]. It also set an ambitious target of reducing new HIV incidence by 75% with the launch of the Kenya AIDS Strategic Framework 2014/15−2018/19 [16]. However, the country was not able to achieve its HIV prevention targets by 2019 and has reprioritized reducing new HIV acquisitions as one of the objectives of its newest framework, the Kenya AIDS Strategic Framework II, 2019/20−2024/25 [17]. To this end, Kenya's National AIDS and STI Control Programme (NASCOP) and the National Syndemic Diseases Control Council (NSDCC), in partnership with the University of Manitoba, conducted a sub‐national epidemic appraisal during 2021−2022 to inform the national HIV prevention strategy. The appraisal was framed around three questions:

1. Which geographies should Kenya prioritize for HIV prevention to achieve the country's goal of new acquisitions reduction by 75%?
2. Which populations should Kenya prioritize in these geographies?
3. What programmes and services should be strengthened and/or scaled up in these geographies and populations?

In this paper, we describe the methods used in the sub‐national epidemic appraisal, present the results from this endeavour and discuss the implications of its findings for Kenya's longer‐term HIV prevention strategy.

## 2 METHODS

The data and methods used in the three components of this epidemic appraisal are described here:

### 2.1 Analysis of HIV prevalence and incidence to identify high‐burden counties for geographic prioritization

For this, we used two measures: (a) the 2021 county‐wise estimates of HIV incidence in populations aged 15 years and above (b) the 2021 county‐wise estimates of HIV prevalence in populations aged 15−49 years. We used HIV incidence and prevalence outputs from three models: the Spectrum/Estimation and Projection Package (EPP) [18] for the national and regional estimates, Naomi (Network‐based Approaches for Modeling HIV Incidence) [19] for county‐level estimates and Shiny90 [20] for the estimates of HIV status awareness. The EPP/Spectrum model, recommended by the UNAIDS Reference Group on Estimates, Modelling and Projections, uses data collected from antenatal clinic surveillance, population‐based surveys including the Kenya AIDS (Acquired Immuno Deficiency Syndrome) Indicator Survey II (KAIS II) and HIV programme data to estimate the prevalence of HIV and AIDS. EPP is used to fit smooth prevalence curves to surveillance and survey data separately for the former eight regions or provinces. The incidence implied by the regional prevalence curves is then transferred to Spectrum where it is combined with additional information on the age structure of incidence and programme coverage (ART, prevention of vertical transmission, cotrimoxazole for children) to estimate indicators such as the number of people living with HIV, the number of new acquisitions, AIDS deaths and the need for ART, prevention of vertical transmission and cotrimoxazole. These regional estimates, along with the number of persons living with HIV (PLHIV), are then exported to the Naomi model to obtain the county‐level estimates of incidence, prevalence and other estimates. Although the Spectrum/EPP/Naomi model is the only source of county‐wise annual estimates of HIV incidence and prevalence, like any other modelling approach, the model outputs are affected by data quality and assumptions. Naomi model, in particular, tends to underestimate the incidence and prevalence among children in Kenya.

### 2.2 Analysis of population size and HIV prevalence to define epidemic typology and prioritize populations for preventive programmes

In order to determine which populations—key, bridging or general populations (GPs)—need to be prioritized for HIV prevention, three measures—HIV prevalence in the GP (same as in 2.1 (b) above), HIV prevalence among the key populations (KPs) and density of KPs per 1000 adult men aged 15−64 years were used, in that order, for the classification of counties into epidemic typology. We used Spectrum/EPP/Naomi model estimates of HIV prevalence in the GP, the KP size estimates of 2020 [21], number of men aged 15−64 years as per the population census of 2019 and estimates of HIV prevalence in KPs (female sex workers [FSWs], men who have sex with men [MSM] and persons who inject drugs [PWID]) based on self‐reported HIV status from the Polling Booth Surveys [22]. The 2020 estimation of KPs, led by NASCOP, was conducted using several methods, including unique object multiplier, unique event multiplier, three source capture‐recapture, service multiplier, successive sampling population size estimation and anchored multiplier. Multiple indicator regression was used to estimate the population sizes for the counties, including those not included in the primary data collection. Stakeholder consensus meetings were conducted to build consensus and validate the size estimates. Though various methods were used for estimating the size of the KP, countries where sex work or same sex relationships are criminalized like Kenya, it is always challenging to find accurate estimates. In Kenya, the population size estimates of MSM is below the global target [23]. Polling booth survey is a group interview method where the individual participants give their responses through a ballot box, thus keeping the individual responses anonymous and unlinked. The details of the method are described elsewhere [24].

**Figure 1:**
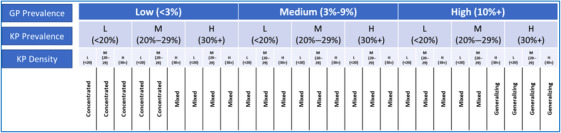
**Algorithm used for epidemic typology classification of the counties (L = Low, M = Medium and H = High)**. Abbreviations: GP, general populations; KP, key populations.

Figure 1 provides the algorithm used for the classification of counties into three epidemic typologies. First, counties were grouped into three groups, based on the distribution of counties according to HIV prevalence in the GP: low (<3%), medium (3−10%) and high (>10%). Counties in each of these groups were further classified into three groups based on the distribution of counties according to HIV prevalence in any of the KPs (i.e. FSW, MSM and PWID): low (<20%), medium (20−29%) and high (30%+), resulting in a total of nine groups. Lastly, counties in each of the nine groups were further divided into three groups, based on the density of total KP (i.e. sum of the estimated number of FSW, MSM and PWID divided by the number of adult men): low (<20 KPs per 1000 adult men), medium (20−29 KPs per 1000 adult men) and high (30+ KPs per 1000 adult men). The first five subgroups with low GP prevalence plus low/medium KP prevalence plus low/medium KP density were classified as concentrated epidemics. The last four groups with high GP prevalence plus high KP prevalence and any categories of KPs per 1000 men were classified as generalized epidemics. The groups in between were classified as mixed epidemics.

### 2.3 Analysis of routine programme monitoring data to assess programme coverage

Lastly, the epidemic appraisal included an analysis of routine programme monitoring data to assess the programme coverage gaps. We analysed county‐wise crude contact coverage gaps across a range of HIV prevention programmes including prevention of vertical transmission (called Prevention of Mother to Child Transmission [PMTCT] programme in Kenya), anti‐retroviral therapy (ART), KP programmes, adolescent girls and young women (AGYW) and VMMC. We used routine programme monitoring data for the year 2021. Table 1 provides details of the coverage indicators used for the different HIV prevention programmes assessed.

**Table 1:** Details of the indicators used to measure coverage gaps in different HIV prevention programmes

Programme	Coverage indicator	Numerator (source)	Denominator (source)
PMTCT programme	% of estimated pregnant women tested for HIV	# of pregnant women tested for HIV (KHIS, January−December 2021)	Estimated # of pregnant women (Spectrum/EPP/Naomi model estimates for 2021)
	% of estimated HIV‐positive pregnant women on ART	# of HIV‐positive pregnant women on ART (KHIS, January−December 2021)	Estimated HIV‐positive pregnant women (Spectrum/EPP/Naomi model estimates for 2021)
ART programme	% of PLHIV on ART	# of persons on ART in 2021 (KHIS)	# of PLHIV (Spectrum/EPP/Naomi Model estimates for 2021)
Key population programme (separately for FSWs, MSM and PWID)	% of FSW/MSM/PWID who received at least one service in the last quarter	# of FSW/MSM/PWID who received at least one service in the last quarter (KHIS, quarter ending December 2021)	# of FSW/MSM/PWID (Key population size estimation report, 2020)
AGYW programme	% of women and girls aged 15−24 years tested for HIV in the year	# of women and girls aged 15−24 years tested for HIV in the year (KHIS, 2021)	# of women and girls aged 15−24 in need of HIV prevention services (estimated by UNAIDS in 2022) [25]
VMMC programme	% of men and boys who underwent circumcision in the year	# of men and boys who underwent circumcision (KHIS, 2021)	# of men and boys uncircumcised (estimated based on the—KENPHIA 2018)

Abbreviations: AGYW, adolescent girls and young women; ART, Anti‐retroviral therapy; FSWs, female sex workers; KENPHIA, Kenya Population‐based HIV Impact Assessment; KHIS, Kenya Health Information System; MSM, men who have sex with men; PLHIV, people living with HIV; PMTCT, Prevention of Mother to Child Transmission; PWID, people who inject drugs; VMMC, voluntary medical male circumcision; #, number.

The quality of routine monitoring data used for measuring prevention programme coverage gaps is expected to vary across counties in terms of accuracy, completeness and consistency [26, 27].

### 2.4 Ethics approval

The study used secondary data that was made available by NSDCC and NASCOP. No primary data were collected for this study. Data were extracted from already published reports, estimates and routine programme data. Data used in the study cannot be linked to any specific individual. Hence, ethics approval was not sought specifically for this study.

## 3 RESULTS

### 3.1 HIV incidence and prevalence

**Figure 2:**
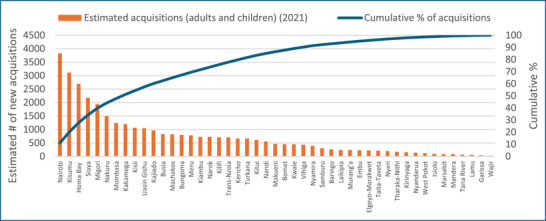
**County‐wise number of new HIV acquisitions and cumulative percentage of new HIV acquisitions, Kenya, 2021**.

Ten counties with more than 1000 new acquisitions accounted for 57% of new acquisitions in Kenya in 2021 (Figure 2). Five of these “high burden” counties—Nairobi, Kisumu, Homa Bay, Siaya and Migori—with more than 1500 new acquisitions per year, contributed to 40% of all new acquisitions.

### 3.2 Epidemic typology

As presented in Table 2, 24 counties were grouped into concentrated epidemic type, due to their low prevalence in the GP, high prevalence in KPs and relatively higher density of FSW and MSM populations. Four counties reflected a generalized epidemic, where HIV prevalence was more than 10% and 30%, respectively, among the general and key populations. The remaining 19 counties were classified as having mixed epidemics.

**Table 2:** County‐wise epidemic typology based on HIV prevalence in general and key populations (KPs), and density of key populations, Kenya, 2021

		HIV prevalence in KPs			KP size estimation				
County	HIV prevalence in the general population	FSW %	MSM %	PWID %	FSW *n*	MSM *n*	PWID *n*	*n* of KP per 1000 adult men	Epidemic typology
Baringo	1.65	20.6	9.5	18.8	1970	568	339	9	Concentrated
Bomet	2.40	22.0	8.7	19.1	2603	409	582	9	Concentrated
Bungoma	2.45	21.6	11.5	19.0	3716	1353	413	8	Concentrated
Elgeyo‐Marakwet	2.02	23.0	8.7	19.3	1268	186	291	5	Concentrated
Embu	2.17	21.8	8.8	19.1	1851	427	0	12	Concentrated
Garissa	0.17	18.0	10.6	18.3	2149	1285	299	15	Concentrated
Isiolo	1.85	21.1	9.0	18.9	688	346	817	5	Concentrated
Kiambu	2.27	19.5	21.7	18.6	5809	2580	1045	20	Concentrated
Laikipia	2.23	20.8	9.2	18.9	1182	231	375	4	Concentrated
Lamu	2.26	21.7	8.9	19.0	749	211	450	4	Concentrated
Machakos	3.02	23.0	13.4	19.3	4932	2811	40	11	Concentrated
Makueni	2.80	24.0	9.2	19.4	2743	893	399	10	Concentrated
Mandera	0.41	18.3	10.2	18.4	3952	1052	519	6	Concentrated
Marsabit	0.86	19.7	9.2	18.6	1530	476	392	4	Concentrated
Meru	2.54	23.0	8.6	19.3	2743	1026	60	14	Concentrated
Murang'a	2.41	22.3	10.0	19.1	2532	904	412	7	Concentrated
Nandi	2.79	21.7	9.4	19.0	2957	514	661	9	Concentrated
Narok	2.88	25.0	8.6	19.7	3107	704	403	9	Concentrated
Nyandarua	2.00	21.1	9.1	18.9	1785	403	257	7	Concentrated
Nyeri	2.97	25.0	9.3	19.7	1317	406	0	8	Concentrated
Tana River	1.05	19.5	8.7	18.6	1798	212	524	43	Concentrated
Tharaka‐Nithi	2.52	21.4	8.8	19.0	2594	219	568	9	Concentrated
Wajir	0.16	18.3	9.9	18.4	3139	860	0	7	Concentrated
West Pokot	0.83	19.8	8.9	18.7	2304	281	617	8	Concentrated
Trans‐Nzoia	3.39	24.0	10.3	19.4	3147	1114	180	8	Mixed
Turkana	3.10	28.0	9.4	20.2	3722	515	609	20	Mixed
Uasin Gishu	3.95	26.0	11.2	19.8	2886	1693	676	18	Mixed
Vihiga	4.58	25.6	9.5	19.8	1940	203	407	10	Mixed
Busia	5.44	32.0	9.6	21.1	2421	550	281	6	Mixed
Kajiado	3.53	25.0	11.3	19.6	7645	1759	436	20	Mixed
Kakamega	3.58	24.0	10.6	19.4	3525	1378	329	3	Mixed
Kericho	3.24	23.0	9.5	19.3	2333	605	180	3	Mixed
Kilifi	2.78	21.3	14.0	19.0	6696	4589	3168	35	Mixed
Kirinyaga	2.51	23.0	8.5	19.2	2497	437	381	31	Mixed
Kisii	4.66	27.0	9.4	20.0	6538	885	29	15	Mixed
Kitui	3.29	26.0	9.5	19.9	2972	500	387	15	Mixed
Kwale	3.11	24.0	9.8	19.5	2833	1026	1127	19	Mixed
Mombasa	5.37	26.0	14.0	19.9	8187	3117	1992	104	Mixed
Nairobi	4.32	23.0	28.2	19.4	39,227	15,271	4198	148	Mixed
Nakuru	3.46	22.3	12.5	19.1	17,708	2706	9	126	Mixed
Nyamira	3.75	24.0	8.7	19.4	1999	193	654	21	Mixed
Samburu	4.59	20.7	8.6	18.8	1500	150	488	12	Mixed
Taita‐Taveta	3.46	25.0	8.7	19.8	1843	219	514	7	Mixed
Homa Bay	16.18	46.1	9.0	23.8	3823	983	55	15	Generalized
Kisumu	15.47	43.1	13.3	23.2	5277	4025	390	59	Generalized
Migori	10.38	36.6	9.6	21.9	5238	782	153	9	Generalized
Siaya	14.06	39.9	9.8	22.6	3724	593	567	11	Generalized

Abbreviations: FSW, female sex workers; MSM, men who have sex with men; PWID, people who inject drugs.

### 3.3 Programme gaps

A summary of county‐wise programme coverage gaps along with the diseases burden and epidemic typology is provided in Table S1.

#### 3.3.1 PMTCT programme

**Figure 3:**
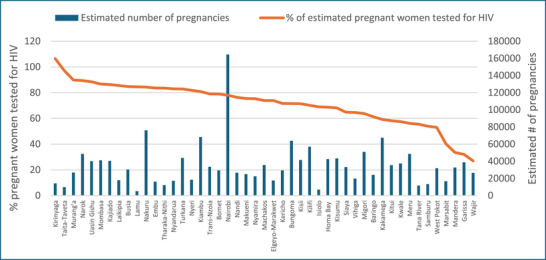
**County‐wise percentage of pregnant women tested for HIV and the estimated number of pregnancies, Kenya, 2021**.

**Figure 4:**
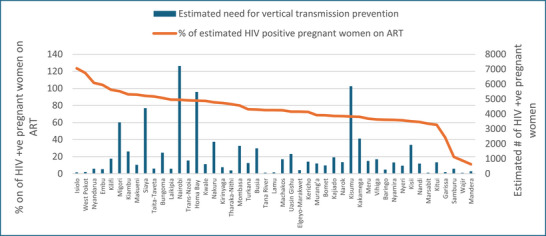
**County‐wise percentage of HIV‐positive pregnant women on anti‐retroviral therapy (ART) and the estimated number of HIV‐positive pregnant women, Kenya, 2021**.

Overall, an estimated 72% of the pregnant women in Kenya had undergone HIV testing in 2021 (Figure 3). The estimated HIV testing rate among pregnant women fell below the national average in 19 counties. In four counties (Marsabit, Mandera, Garissa and Wajir), less than half of pregnant women underwent HIV testing at antenatal care. Seventy‐nine percent of the pregnant women with PMTCT need in the country were on ART (Figure 4). More than 80% of the estimated women with PMTCT need were on ART in 19 (40% of the) counties. Four counties—Garissa, Samburu, Wajir and Mandera—had lower than 50% of the estimated HIV‐positive pregnant women on ART.

#### 3.3.2 ART

**Figure 5:**
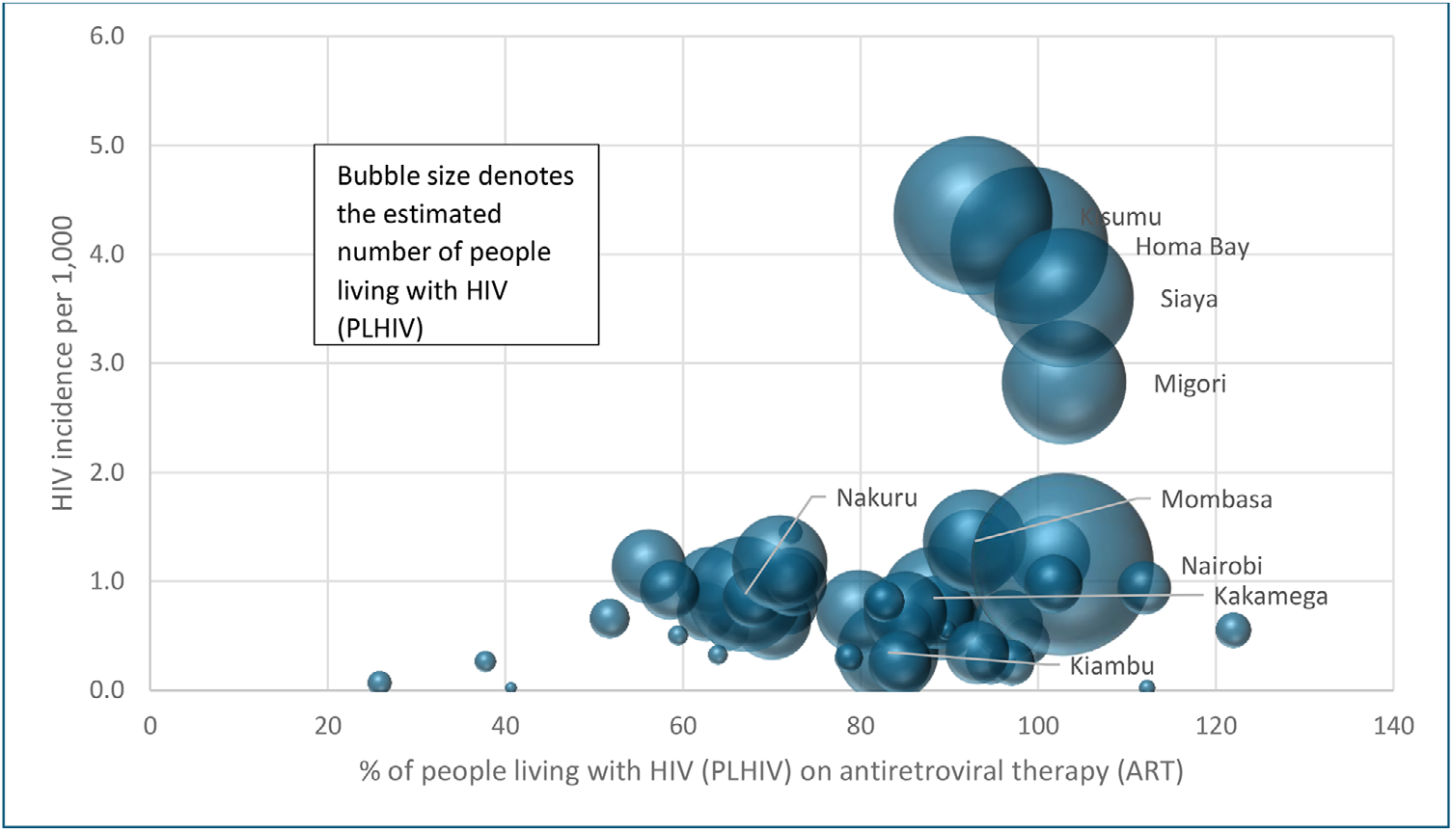
**County‐wise percentage of persons living with HIV (PLHIV) on anti‐retroviral therapy (ART) according to HIV incidence and estimated number of PLHIV, Kenya, 2021**.

During 2021, 89% of Kenya's estimated number of PLHIV were on ART, and the ART coverage ranged from 26% in Mandera to 122% in Tharaka Nithi. Of the nine counties with more than 50,000 estimated PLHIV, the ART coverage ranged from 67% in Nakuru (estimated PLHIV of 65,860) to over 100% each in Migori (PLHIV estimate of 76,884), Nairobi (PLHIV estimate of 165,903) and Siyaya (PLHIV estimate of 96,578) (Figure 5). Likely explanations for when ART coverage was more than 100% of the estimated number of PLHIV in a county include PLHIV from outside the county accessing ART services, and duplication of some individuals.

#### 3.3.3 Key population programme

**Figure 6:**
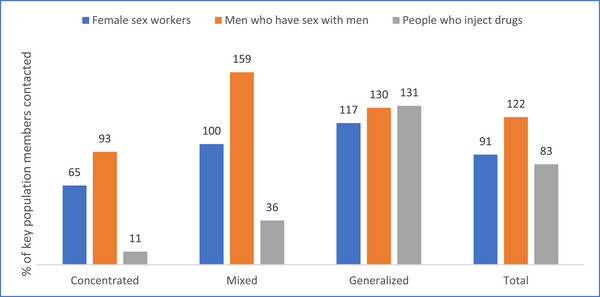
**Percentage of key population (KP) members contacted by KP programmes according to epidemic typology of the county, Kenya, 2021**.

During October−December 2021, the KP programmes reached 91% of the 197,096 estimated FSW, 122% of the estimated 61,650 MSM and 83% of the estimated 27,056 PWID. The contact coverage for all three KP programmes is the lowest among the 24 counties with concentrated epidemic. While the FSW and PWID contact coverage was the highest among the four counties with the generalized epidemic, it was the highest for the MSM in the 19 counties with mixed epidemic (Figure 6). More than 100% coverage could probably be due to double counting of MSM in different programmes. It is most likely that the MSM population in Kenya is underestimated [28].

**Figure 7:**
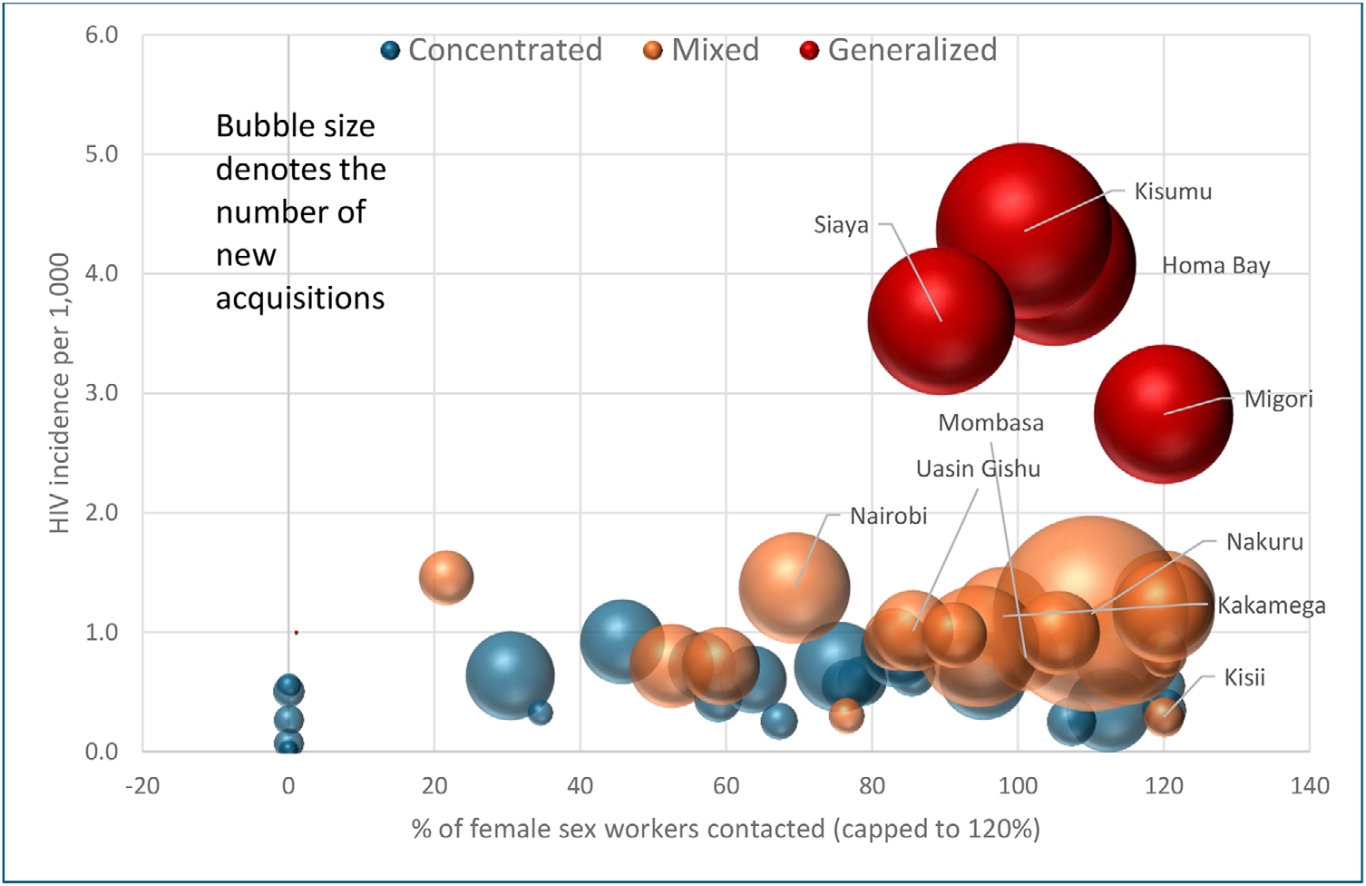
**County‐wise percentage of female sex workers contacted by key population programmes according to HIV incidence per 1000, epidemic typology and the number of new HIV acquisitions, Kenya, 2021**. *Note*: Only the names of counties with 1000+ new HIV acquisitions are displayed in the figure.

In six of the 24 concentrated epidemic counties, none of the estimated FSWs were contacted during the last quarter of 2021 and these six counties had fewer than 100 new HIV acquisitions in 2021 (Figure 7). All the 19 mixed epidemic counties had FSW programmes, of which four had contact coverage of <80%, of which three counties (Mombasa, Kilifi and Kitui) had >500 new acquisitions in 2021. All generalized epidemic counties, which also had higher HIV incidence (>3 per 1000) had FSW contact coverage of over 80%.

**Figure 8:**
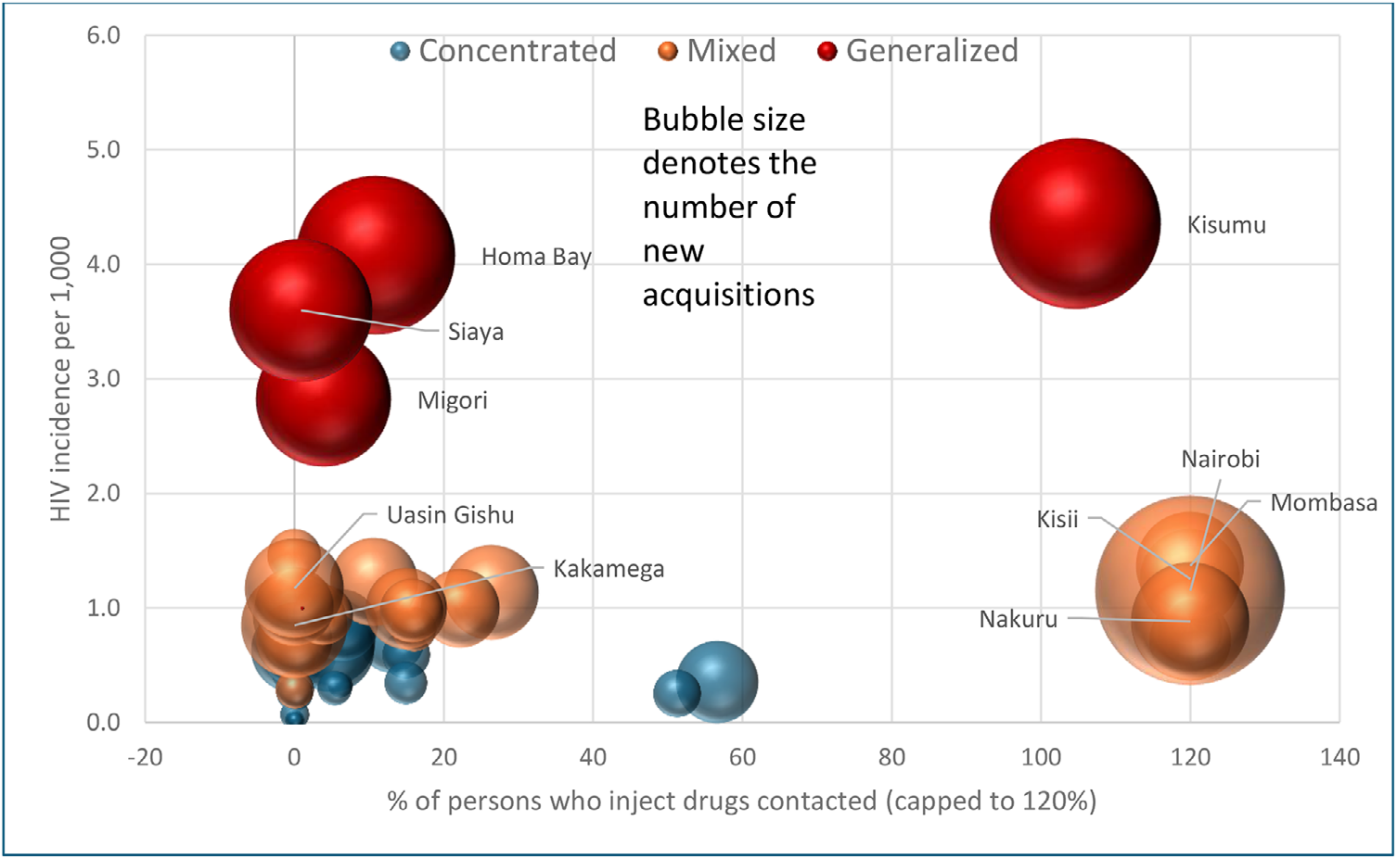
**County‐wise percentage of people who inject drugs contacted by key population programme according to HIV incidence per 1000, epidemic typology and the number of new HIV acquisitions, Kenya, 2021**. *Note*: Only the names of counties with 1000+ new HIV acquisitions are displayed in the figure.

Only 10 of the 24 concentrated epidemic counties and 13 of the 19 mixed epidemics had PWID programmes during 2021. None of the concentrated epidemic counties with PWID programmes has achieved a contact coverage of 80%. Among the 13 counties with mixed epidemics that had a PWID programme, six had achieved a contact coverage of 80% or more. Except Kisumu, all generalized epidemic counties, which also had higher HIV incidence (>3 per 1000) had PWID contact coverage of <20% (Figure 8). Except Kiambu, which had a contact coverage of 57%, all the five counties with an estimated 1000+ PWID had over 100% contact coverage (data not shown).

#### 3.3.4 AGYW programme

**Figure 9:**
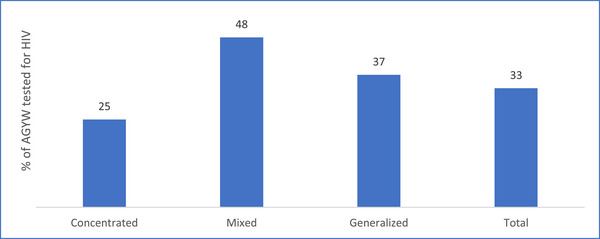
**Percentage of adolescent girls and young women (AGYW) tested for HIV, according to epidemic typology of the county, Kenya, 2021**.

**Figure 10:**
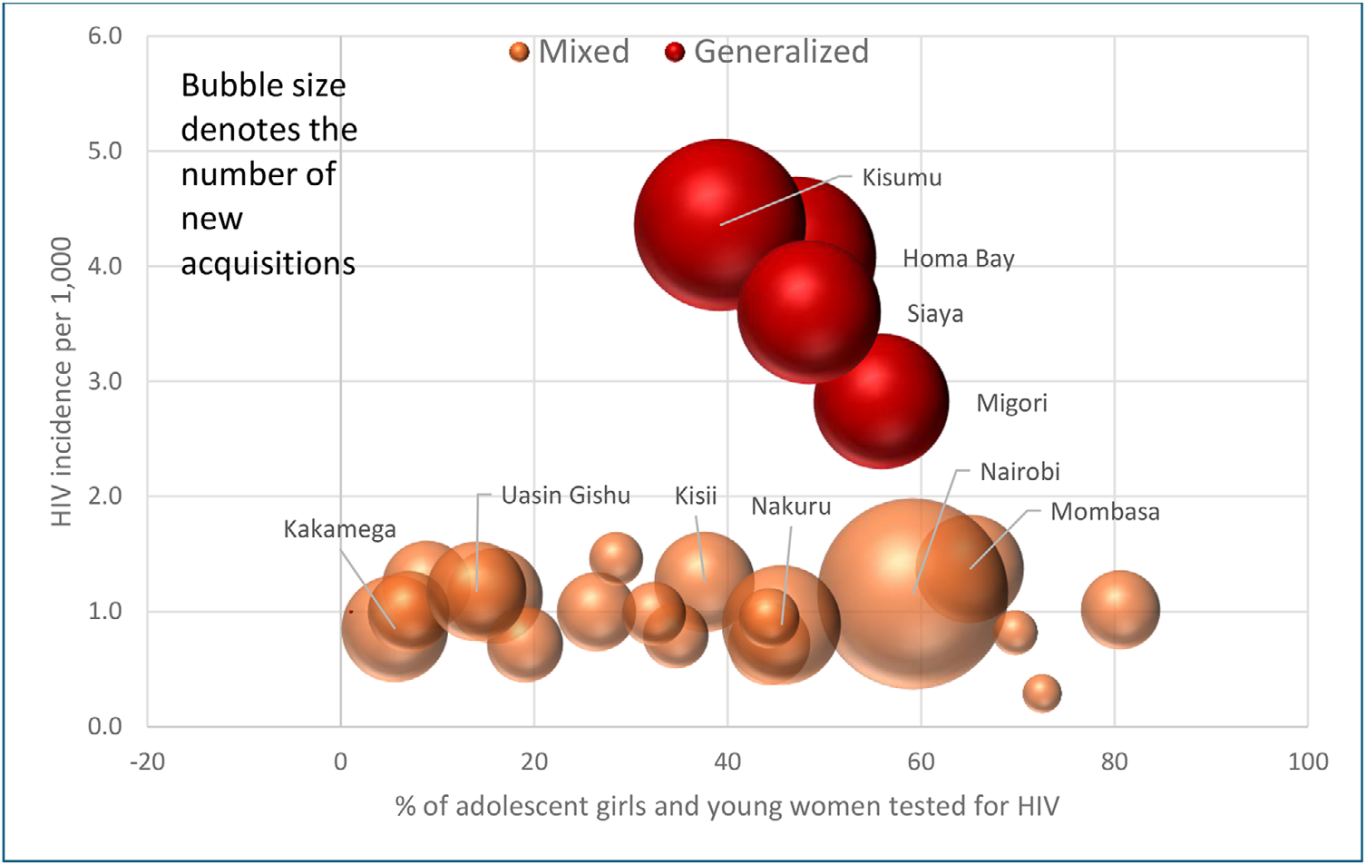
**County‐wise percentage of adolescent girls and young women tested for HIV among mixed and generalized epidemics according to the number of new acquisitions, Kenya, 2021**. *Note*: Only the names of counties with 1000+ new HIV acquisitions are displayed in the figure.

Overall, a third of the estimated AGYW aged 15−24 years in need of HIV prevention services were tested for HIV in 2021. The HIV testing coverage among the AGYW was at 37% in the four counties with generalized epidemic and 48% in the 19 counties with mixed epidemic (Figures 9 and 10). Among the generalized epidemic counties, Kisumu with the largest estimated new acquisitions had the lowest AGYW coverage of only 39%.

#### 3.3.5 VMMC programme

**Figure 11:**
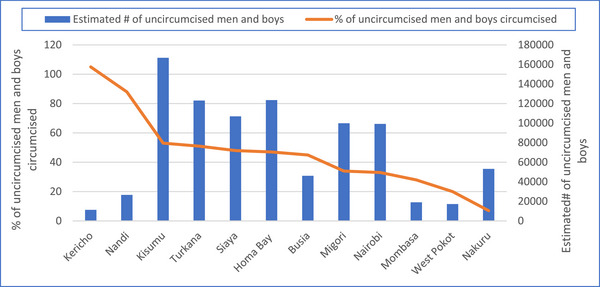
**County‐wise percentage of uncircumcised men and boys circumcised and the estimated number of uncircumcised men and boys among counties with voluntary medical male circumcision (VMMC) programme, Kenya, 2021**.

The VMMC programme in Kenya focuses on 12 priority counties. These include five culturally non‐circumcising counties, Turkana, Kisumu, Migori, Siaya and Homa Bay, and seven culturally circumcising counties with non‐circumcising subgroups, Mombasa, Nairobi, Busia, West Pokot, Nandi, Nakuru and Kericho. According to KHIS 2021, 44% of the estimated 893,057 uncircumcised men and boys in these counties were circumcised. The VMMC coverage was greater than 80% in two counties (Kericho and Nandi) with relatively fewer estimated needs (Figure 11).

## 4 DISCUSSION

This study sought to use available data to systematically prioritize the sub‐national geographies, populations and programmes for achieving the Government of Kenya's 2030 target of a 75% reduction in new acquisitions. Kenya is one of the few African countries which has emphasized a sub‐national approach in its national strategic plan, by factoring in county‐wise heterogeneity in HIV incidence/prevalence, epidemic typology and gaps in programme coverage. This epidemic appraisal is developed as a tool to support strategic planning and resource allocation—an important sphere of Programme Science. Using the 2021 national and sub‐national HIV incidence and prevalence estimates from Spectrum/EPP/Naomi models, counties with high HIV incidence and prevalence were identified for geographic prioritization. Local epidemic typologies were defined to prioritize populations for HIV prevention programmes. Routine programme monitoring data were analysed to assess coverage gaps in HIV prevention programmes. Characterizing and understanding local epidemiological context to identify priority geographies and populations through the use of different types of available data (e.g. modelling, routine programme monitoring data, population size estimates, bio‐behavioural surveys) is the key feature of this approach.

The HIV burden is unevenly distributed in Kenya, and prioritizing HIV prevention in counties with greater potential for new acquisitions can potentially achieve faster progress. The counties with the largest number of new acquisitions such as Nairobi, Kisumu, Homa Bay, Siaya and Migori need comprehensive HIV prevention programmes.

The population focus for prevention strategies may vary according to the epidemic typology. While the KPs, pregnant women and PLHIV need to be prioritized for HIV prevention in all epidemics, AGYW could be prioritized in generalizing and mixed epidemics and populations such as fisherfolk [29, 30], long‐distance truckers and other similar populations in only generalized epidemics [31].

Prioritization of interventions based not only on disease burden and epidemic typology, but also on the prevailing gaps in coverage is a key aspect of this appraisal. Programme gap analysis helps in identifying counties where certain programmes and services need scaling up and are under‐utilized. In each county, the programmes that are most under‐utilized need to be optimized, depending on its disease burden and epidemic typology. For instance, Nairobi, a mixed epidemic with the largest disease burden in terms of new acquisitions, needs to optimize its AGYW and VMMC programmes. Kisumu, which is a generalized epidemic, and has the second largest new acquisitions, needs to increase coverage of interventions among its KPs, especially the PWID population, populations like fisherfolk, AGYW and men through VMMC. The programme gap analysis will additionally help to generate hypotheses or research questions to learn more, particularly around coverage gaps.

The National Multisectoral HIV Prevention Acceleration Plan 2023−2030 [29] of the Government of Kenya prioritized nine counties with greater disease burden—Nairobi, Kisumu, Homa Bay, Siaya, Migori, Nakuru, Kakamega, Usain Gishu and Kajiado for greater impact. Based on the epidemic typology of a county and sub‐county, the combination of HIV prevention programmes was adjusted and counties were trained in epidemic appraisal at the sub‐county level. While the Acceleration Plan has committed to strengthening outcome measurement, it is too early to measure how these plans have resulted in changes to local epidemic trajectories.

Although an advantage of this approach to sub‐national epidemic appraisal is that it used all secondary data, with no additional data collected for the purpose, its limitation has been the quality of data. Inconsistencies in data collection methods, changes in reporting standards, and variations in data quality across time and sources might have affected the accuracy and comparability of the findings across counties. Contact coverage of over 100%, particularly among the MSM, could largely be due to the underestimation of the size of the MSM population coupled with the possibility of certain populations being double counted, as in some counties, there are multiple implementing partners implementing programmes with MSM. This epidemic appraisal did not evaluate the quality of data—both the Spectrum/EPP/Naomi model estimates as well as routine programme monitoring. While the model estimates are suspected to be less robust at the county level than at the national level, the routine programme monitoring could be subject to under‐reporting and double‐counting that are common to most health management information systems. Data gaps, particularly regarding resource allocations and programme targets, constrained the identification of planned coverage gaps in various HIV prevention programmes.

## 5 CONCLUSIONS

The Government of Kenya's strategic focus on sub‐national planning for HIV prevention led to this novel approach of prioritizing geographies, populations and programmes based entirely on the available data at the county level. The approaches and methods used in this study may be applicable in other countries in Africa and elsewhere. As data are available on an annual basis, this appraisal can be done annually to set goals and targets.

## COMPETING INTERESTS

The authors declare no competing interests.

## AUTHORS’ CONTRIBUTIONS

JB, PB, RBM, SY‐NS and FE designed the epidemic appraisal. JG, JK, FS and PA coordinated data compilation, RBM conducted data analysis and interpretation, RBM, SY‐NS and FE drafted the manuscript, all revised the manuscript, all authors read and approved the final manuscript.

## FUNDING

The Bill & Melinda Gates Foundation funded the study.

## Supporting information


**Table S1**: Summary of county‐wise programme coverage gaps, along with disease burden and epidemic typology, Kenya, 2021

## Data Availability

Data sharing is not applicable to this article as no new data were created in this study.
