# Supplementary material for: A sub‐national HIV epidemic appraisal in Kenya: a new approach for identifying priority geographies, populations and programmes for optimizing coverage for HIV prevention
Source: J Int AIDS Soc. 2024 Jul 10;27(Suppl 2):e26245. doi: 10.1002/jia2.26245 (PMC11233855; doi:10.1002/jia2.26245)
Supplement: Supplementary file 1 — Table S1: Summary of county‐wise programme coverage gaps, along with disease burden and epidemic typology, Kenya, 2021 [file JIA2-27-e26245-s001.docx]

**Supplementary Table 1: Summary of county-wise programme coverage gaps, along with disease burden and epidemic typology, Kenya, 2021**

| County | Population (2019 Census) | Estimated new infections | HIV prevalence (%) in general population age 15-49 | Epidemic typology | % of estimated pregnant women tested for HIV | % of estimated HIV positive pregnant women on ART | % on ART | % of estimated FSW contacted | % of estimated MSM contacted | % of estimated PWID contacted | % AGYW covered | VMMC contact coverage % |
| --- | --- | --- | --- | --- | --- | --- | --- | --- | --- | --- | --- | --- |
| Baringo | 666,763 | 262 | 1.65 | Concentrated | 61 | 63 | 65 | 77 | 12 | 0 | 20 | 0 |
| Bomet | 875,689 | 457 | 2.40 | Concentrated | 79 | 69 | 63 | 57 | 59 | 13 | 29 | 13 |
| Bungoma | 1,670,570 | 796 | 2.45 | Concentrated | 72 | 89 | 89 | 30 | 69 | 0 | 8 | 0 |
| Busia | 893,681 | 828 | 5.44 | Mixed | 85 | 75 | 101 | 121 | 311 | 11 | 9 | 45 |
| Elgeyo-Marakwet | 454,480 | 218 | 2.02 | Concentrated | 74 | 73 | 52 | 85 | 0 | 0 | 9 | 0 |
| Embu | 608,599 | 230 | 2.17 | Concentrated | 84 | 104 | 99 | 59 | 139 | 0 | 38 | 0 |
| Garissa | 841,353 | 38 | 0.17 | Concentrated | 32 | 42 | 112 | 0 | 0 | 0 | 9 | 0 |
| Homa Bay | 1,131,950 | 2695 | 16.18 | Generalized | 69 | 86 | 99 | 105 | 113 | 11 | 47 | 47 |
| Isiolo | 268,002 | 98 | 1.85 | Concentrated | 69 | 124 | 59 | 0 | 0 | 0 | 9 | 2 |
| Kajiado | 1,117,840 | 966 | 3.53 | Mixed | 86 | 68 | 56 | 98 | 79 | 26 | 16 | 1 |
| Kakamega | 1,867,579 | 1198 | 3.58 | Mixed | 59 | 67 | 88 | 115 | 237 | 0 | 6 | 4 |
| Kericho | 901,777 | 666 | 3.24 | Mixed | 72 | 72 | 63 | 103 | 118 | 22 | 26 | 105 |
| Kiambu | 2,417,735 | 729 | 2.27 | Concentrated | 81 | 93 | 83 | 112 | 180 | 57 | 27 | 0 |
| Kilifi | 1,453,787 | 713 | 2.78 | Mixed | 70 | 99 | 80 | 53 | 76 | 178 | 44 | 3 |
| Kirinyaga | 610,411 | 157 | 2.51 | Mixed | 106 | 83 | 95 | 138 | 287 | 0 | 73 | 0 |
| Kisii | 1,266,860 | 1065 | 4.66 | Mixed | 71 | 61 | 93 | 126 | 255 | 159 | 38 | 0 |
| Kisumu | 1,155,574 | 3117 | 15.47 | Generalized | 68 | 67 | 93 | 101 | 129 | 105 | 39 | 53 |
| Kitui | 1,136,187 | 614 | 3.29 | Mixed | 58 | 57 | 89 | 59 | 173 | 0 | 19 | 0 |
| Kwale | 866,820 | 453 | 3.11 | Mixed | 58 | 85 | 72 | 101 | 0 | 241 | 35 | 1 |
| Laikipia | 518,560 | 243 | 2.23 | Concentrated | 86 | 87 | 85 | 79 | 86 | 15 | 39 | 1 |
| Lamu | 143,920 | 58 | 2.26 | Concentrated | 85 | 75 | 90 | 0 | 0 | 0 | 38 | 0 |
| Machakos | 1,421,932 | 821 | 3.02 | Concentrated | 74 | 74 | 85 | 76 | 56 | 5 | 20 | 0 |
| Makueni | 987,653 | 464 | 2.80 | Concentrated | 75 | 93 | 97 | 64 | 37 | 0 | 31 | 15 |
| Mandera | 867,457 | 88 | 0.41 | Concentrated | 34 | 11 | 26 | 0 | 0 | 0 | 10 | 0 |
| Marsabit | 459,785 | 89 | 0.86 | Concentrated | 40 | 59 | 38 | 0 | 0 | 0 | 5 | 1 |
| Meru | 1,545,714 | 782 | 2.54 | Concentrated | 56 | 65 | 70 | 95 | 162 | 5 | 30 | 0 |
| Migori | 1,116,436 | 1943 | 10.38 | Generalized | 64 | 97 | 103 | 162 | 347 | 4 | 56 | 34 |
| Mombasa | 1,208,333 | 1241 | 5.37 | Mixed | 87 | 80 | 93 | 69 | 216 | 128 | 65 | 28 |
| Murang'a | 1,056,640 | 239 | 2.41 | Concentrated | 90 | 69 | 84 | 107 | 324 | 51 | 34 | 1 |
| Nairobi City | 4,397,073 | 3828 | 4.32 | Mixed | 78 | 87 | 103 | 110 | 108 | 232 | 59 | 33 |
| Nakuru | 2,162,202 | 1496 | 3.46 | Mixed | 84 | 84 | 67 | 95 | 183 | 178 | 46 | 7 |
| Nandi | 885,711 | 555 | 2.79 | Concentrated | 76 | 61 | 68 | 84 | 7 | 6 | 26 | 88 |
| Narok | 1,157,873 | 725 | 2.88 | Concentrated | 89 | 68 | 59 | 46 | 77 | 0 | 43 | 0 |
| Nyamira | 605,576 | 391 | 3.75 | Mixed | 75 | 63 | 112 | 83 | 107 | 4 | 44 | 0 |
| Nyandarua | 638,289 | 137 | 2.00 | Concentrated | 83 | 107 | 97 | 67 | 109 | 0 | 48 | 0 |
| Nyeri | 759,164 | 193 | 2.97 | Concentrated | 82 | 63 | 93 | 211 | 329 | 15 | 38 | 2 |
| Samburu | 310,327 | 307 | 4.59 | Mixed | 54 | 20 | 72 | 22 | 0 | 0 | 28 | 1 |
| Siaya | 993,183 | 2180 | 14.06 | Generalized | 65 | 91 | 103 | 90 | 193 | 1 | 48 | 48 |
| Taita-Taveta | 340,670 | 209 | 3.46 | Mixed | 97 | 91 | 83 | 147 | 366 | 16 | 70 | 17 |
| Tana River | 315,943 | 65 | 1.05 | Concentrated | 56 | 75 | 64 | 34 | 0 | 6 | 29 | 0 |
| Tharaka-Nithi | 393,177 | 175 | 2.52 | Concentrated | 84 | 82 | 122 | 158 | 736 | 0 | 32 | 0 |
| Trans-Nzoia | 990,341 | 709 | 3.39 | Mixed | 79 | 86 | 72 | 106 | 95 | 15 | 7 | 1 |
| Turkana | 926,976 | 665 | 3.10 | Mixed | 83 | 76 | 72 | 86 | 157 | 0 | 81 | 51 |
| Uasin Gishu | 1,163,186 | 1048 | 3.95 | Mixed | 88 | 73 | 71 | 138 | 94 | 0 | 14 | 0 |
| Vihiga | 590,013 | 434 | 4.58 | Mixed | 65 | 64 | 102 | 91 | 133 | 16 | 32 | 0 |
| Wajir | 781,263 | 27 | 0.16 | Concentrated | 27 | 16 | 41 | 0 | 0 | 0 | 6 | 0 |
| West Pokot | 621,241 | 126 | 0.83 | Concentrated | 53 | 118 | 79 | 76 | 0 | 5 | 29 | 20 |
